# The Relation between Inflammation and Neuropsychological Test Performance

**DOI:** 10.1155/2012/703871

**Published:** 2012-09-13

**Authors:** Valerie H. Balldin, James R. Hall, Robert C. Barber, Linda Hynan, Ramon Diaz-Arrastia, Sid E. O'Bryant

**Affiliations:** ^1^Psychology Service, South Texas Veterans Healthcare System, San Antonio, TX 78229, USA; ^2^Department of Psychiatry & Behavioral Health, University of North Texas Health Science Center, Fort Worth, TX 76107, USA; ^3^Department of Pharmacology & Neurosicence, University of North Texas Health Science Center, Fort Worth, TX 76107, USA; ^4^Department of Clinical Sciences, University of Texas Southwestern Medical Center, Dallas, TX 75390, USA; ^5^Department of Neurology and Neurotherapeutics, University of Texas Southwestern Medical Center, Dallas, TX 75390, USA; ^6^Department of Internal Medicine, University of North Texas Health Science Center, Fort Worth, TX 76107, USA; ^7^Institute for Aging and Alzheimer's Disease Research, University of North Texas Health Sciences Center, Fort Worth, TX 76107, USA

## Abstract

*Background*. Considerable research documents an association between pro- and anti-inflammatory markers and Alzheimer's disease (AD), yet the differential relation between these markers and neuropsychological functioning in AD and nondemented controls has received less attention. The current study sought to evaluate the relationship between peripheral markers of inflammation (both pro- and anti-inflammatory) and neuropsychological functioning through the Texas Alzheimer's Research and Care Consortium (TARCC) cohort. *Methods*. There were 320 participants (Probable AD *n* = 124, Controls *n* = 196) in the TARCC Longitudinal Research Cohort available for analysis. Regression analyses were utilized to examine the relation between proinflammatory and anti-inflammatory markers and neuropsychological functioning. Follow-up analyses were conducted separately by case versus control status. *Results*. Proinflammatory and anti-inflammatory markers were found to be associated with neuropsychological testing. Third tertile proinflammatory markers were negatively associated with measures of attention and language, and anti-inflammatory markers were positively associated with measures of immediate verbal memory and delayed verbal and visual memory. *Conclusions*. These findings support the link between peripheral inflammatory markers and neuropsychological functioning and suggest the utility of examining profiles of inflammatory markers in the future.

## 1. Introduction

Inflammation has been hypothesized to modulate a number of pathogenic processes that are associated with Alzheimer's disease (AD), including the amyloid cascade [1–3], although the underlying etiology and full nature of the relationship remains unclear. Both proinflammatory and anti-inflammatory proteins have been shown to be stimulated by injury, *β*-amyloid toxicity, and ischemia [4], and inflammation is increased in both hypertension and atherosclerosis [5]. Furthermore, peripheral inflammation has been related to poorer cognitive performance [6–9], as well as cognitive decline [10], although these findings have not always been consistently supported [11]. 

Several large population-based studies have examined serum markers of inflammation and their relation to cognitive functioning. The Honolulu Asia Aging Study (HAAS) [12] found that raised levels of C-reactive protein (CRP; an inflammatory marker) in mid-life was associated with a significantly increased risk for vascular dementia (VaD) and AD, with or without the presence of cardiovascular disease (CVD). The Rotterdam Study reported a link between CRP risk for AD and VaD. Additionally, it has been proposed that individuals with cognitive impairment may have a different balance of proinflammatory and anti-inflammatory cytokines compared to those with normal aging [13, 14]. 

While considerable research has documented that chronic inflammation and high levels of proinflammatory cytokines are fundamental components of AD, the relationship between these markers and decline in specific neuropsychological performance has received less attention. Research in this area has tended to use brief measures of global cognitive functioning (e.g., mini-mental state examination (MMSE), Short Portable Mental Questionnaire (SPMQ) making it difficult to gain insight into focal cognitive deficits that may be associated with elevated cytokine levels [15]. 

The current project sought to examine the link between a number of pro- and anti-inflammatory markers and neuropsychological functioning among a sample of patients diagnosed with AD and cognitively normal controls from the Texas Alzheimer's Research and Care Consortium (TARCC) research. It was hypothesized that there would be a significant relationship between serum levels of inflammatory cytokines (a net increase in the proinflammatory profile) and performance on tests of memory and verbal fluency compared to other neuropsychological domains. 

## 2. Materials and Methods

**Participants**

Participants included 320 individuals (124 diagnosed with Probable AD and 196 normal controls) enrolled the TARCC Longitudinal Research Cohort. The methodology of the TARCC project has been described in detail elsewhere [16]. Briefly, each participant undergoes an annual evaluation that includes a medical examination, interview, neuropsychological testing, and blood draw. All patients met consensus-based diagnosis for probable AD based on NINCDS-ADRDA criteria [17] and controls performed within normal limits on psychometric assessment and were assigned a Clinical Dementia Rating scale (CDR) global score of 0.0. AD patients were included if their CDR global scores were .5 or 1.0; 46 had CDR global scores of 0.5 and 78 had scores of 1.0. The TARCC project received institutional review board approval and all participants and/or caregivers provided written informed consent.

## 3. Measures

The TARCC neuropsychology core battery consists of neuropsychological instruments administered as part of the established Alzheimer's disease clinical/research platforms at each participating institution and included digit span (WAIS-R, WAIS-III, WMS-R), Trail Making Test, WMS Logical Memory (WMS-R and WMS-III), Boston Naming Test (30- and 60-item versions), verbal fluency (FAS), Clock Drawing Test, the American National Adult Reading Test (AMNART), the Geriatric Depression Scale (GDS-30), mini-mental state examination (MMSE) [18], and the Clinical Dementia Rating scale (CDR) [19]. In order to equate scores from digit span and story memory scales, all raw scores were converted to scale scores based on previously published normative data [20]. For the Boston Naming Test, the current group recently conducted an independent study that demonstrated the psychometric properties of an estimated 60-item BNT score that can be calculated from 30-item versions [21]. Adjusted scale scores were utilized as dependent variables in analyses.

## 4. Assays

Nonfasting samples were collected in serum-separating tubes during clinical evaluations, allowed to clot at room temperature for thirty minutes, centrifuged, aliquoted, and stored at −80°C in plastic vials. Serum samples were sent frozen to Rules-Based Medicine (http://www.rulesbasedmedicine.com/, Austin, TX, USA) where they were thawed for assay without additional freeze-thaw cycles. Rules-Based Medicine conducted multiplexed immunoassay via their human multianalyte profile (human MAP). Multiple proteins, including proinflammatory markers (TNFalpha, Alpha1Antitrypsin, IL-8, and C-reactive protein) and anti-inflammatory markers (IL-1ra, IL-10), were quantified though multiplex fluorescent immunoassay utilizing colored microspheres with protein-specific antibodies. The coefficient of variation (CV), a normalized measure of dispersion of a probability distribution (CV= standard deviation/mean), for each marker (run only once) are shown in Table 1. The proinflammatory and anti-inflammatory markers were chosen due to past research showing their reliability in use in the study of inflammatory processes [22–26].

Additional information regarding assay performance can be found online. Assays conducted by this company utilizing this platform, including TARCC data, have been published elsewhere [27–29]. For additional information, please refer to (http://www.rulesbasedmedicine.com/). 

## 5. Analyses

Statistical analyses were conducted using SPSS version 18.0 (SPSS, Chicago, IL). Comparisons between NC and AD were conducted using *t*-tests for continuous measures and *χ* ^2^ for categorical measures. The relation between inflammatory makers and cognitive test performance was examined via a series of regression models for all participants (AD and NC) with proinflammatory and anti-inflammatory proteins entered in separate blocks as predictor variables and neuropsychological test scores entered as outcome variables. Levels of inflammatory proteins were categorized into tertiles based on the distribution of the variable (i.e., 1st, 2nd, and 3rd group). The number of markers (pro- and anti-inflammatory separately) in the third tertile for each marker was summed for each participant and this summed score was entered as a predictor variable into a linear regression model with neuropsychological test scores as outcome variables (i.e., if the participant's value for IL-8 and IL-10 were in the third tertile, the participant had a “2” entered as the predictor variable into the regression model). 

Follow-up analyses were then conducted examining the relationship between proinflammatory and anti-inflammatory markers and neuropsychological functioning on case status (NC versus AD). All biomarker results were transformed using log base 10 in order to evenly distribute the scores of the markers. Assumptions for all statistics tests were checked for violations and statistical significance was declared for *P* value <0.01. 

## 6. Results

Average age and education of the control group was 70.5 ± 8.8 and 15.5 ± 2.7, respectively, while average age and education of the AD group was 76.7 ± 7.8 and 14.4 ± 3.3, respectively. The sample was over 95% Caucasian, and there were more females than males, though gender distribution was not significantly different between groups. As expected, AD patients obtained significantly lower MMSE scores (mean = 22.3 ± 4.1, median = 23.0) than controls (mean = 29.4 ± 0.9, median = 30.0) as well as higher CDR sum of boxes scores (AD mean = 5.2 ± 2.3, median = 5; controls mean = 0.0 ± 0.04, median = 0.0). Demographic characteristics of the study population are shown in Table 2.

## 7. Individual Markers and Neuropsychological Functioning

### 7.1. Proinflammatory Markers

Higher IL-8 levels were associated with significantly poorer scores in global cognition (MMSE *β* = −0.185, *P* = 0.002), as well as immediate visual memory (WMS-III Visual Reproduction I *β* = −0.157, *P* = 0.009), and verbal fluency (COWAT *β* = −0.172, *P* = 0.004). Higher IL-8 levels were also associated with significantly greater disease severity (CDR Sum of Boxes; *β* = 0.205, *P* < 0.001).

Higher CRP levels were associated with significantly greater disease severity (CDR Sum of Boxes; *β* = −0.201, *P* = 0.001). However, elevated CRP levels were also related to significantly better global cognition (MMSE; *β* = 0.533, *P* = 0.006), verbal (WMS-III Logical Memory I; *β* = 0.202, *P* = 0.001; WMS-III Logical Memory II; *β* = 0.712, *P* = 0.001), and delayed visual memory (WMS-III Visual Reproduction II; *β* = 0.710, *P* < 0.001).

Higher TNF-alpha levels were associated with better delayed verbal (WMS-III Logical Memory II; *β* = 0.178, *P* = 0.007) and immediate visual memory (WMS-III Visual Reproduction I; *β* = 0.196, *P* = 0.002). Neuropsychological tests by diagnosis are shown in Table 3.

### 7.2. Anti-Inflammatory Markers

Higher IL-1ra levels were associated with disease severity (CDR Sum of boxes; *β* = −0.161, *P* = 0.004), better scores in verbal memory (WMS-III Logical Memory I; *β* = 0.168, *P* = 0.005; WMS-III Logical Memory II; *β* = 0.164, *P* = 0.006), and visual memory (WMS-III Visual Reproduction I; *β* = 0.180, *P* = 0.002; WMS-III Visual Reproduction II; *β* = 0.180, *P* = 0.002). 

## 8. Summed Number of Markers by Tertiles and Separated by Diagnostic Category

The sum of the number of proinflammatory markers in the 3^rd^ tertile was found to be associated with significantly better information processing speed (Trails A; *β* = 0.177, *P* = 0.004). When looking at AD cases only, the sum of the proinflammatory markers was associated with significantly better immediate verbal memory (WMS-III Logical Memory I; *β* = 0.163, *P* = 0.004), but poorer fluency (COWAT; *β* = −0.173, *P* = 0.010). In the NC group, the sum of the proinflammatory markers was associated with significantly better processing speed (Trails A; *β* = 0.165, *P* = 0.010). See Table 4 for biomarkers by diagnosis.

## 9. Discussion

The current study demonstrates that the association between inflammatory markers is quite complex. In our sample, proinflammatory markers were found to be both positively associated with immediate and delayed verbal and visual memory, and disease severity and global cognition and negatively associated with measures of immediate visual memory, verbal fluency, and global cognition. The anti-inflammatory markers were found to be significantly positively and negatively associated with a measure of global cognition and disease severity (CDR), and immediate and delayed visual and verbal memory. These findings are not surprising as such conflicting results have been documented previously in the literature. These findings also make sense biologically as these immune-related markers serve a purpose that only becomes detrimental when the system gets out of balance, which has been observed in AD. Our findings also point to the fact that the relationship between inflammation and neuropsychological functioning varies according to disease status and cognitive domain. In fact, the majority of our significant findings were for AD cases rather than controls. 

When examined by case status, proinflammatory markers were negatively associated with a measure of verbal fluency and positively associated with immediate verbal memory for the AD group. Within the normal control group, proinflammatory markers were positively associated with processing speed. In addition, a positive association with information processing speed was observed among individuals in the 3rd tertile of proinflammatory markers. 

These findings suggest the importance of examining profiles of the inflammatory response. As suggested in prior research, [30] those with cognitive impairment may possess different proinflammatory and anti-inflammatory profiles compared to those without cognitive impairment. For example, the Leiden 85+ study examined the ratio of TNFalpha, a proinflammatory cytokine and IL-10, an anti-inflammatory cytokine. The ratio was higher in patients diagnosed with AD compared with normal controls which suggests a net “proinflammatory profile”. The findings of the current study also indicate the importance of examining the combination of upper median levels of pro- and anti-inflammatory biomarkers. 

The current findings are limited by the cross-sectional nature of the analyses; however, the TARC cohort is being evaluated annually and follow-up analyses examining the profiles of anti- and proinflammatory markers in those with and without a diagnosis of AD will be completed. The current findings point towards the use of profiles of biomarkers as a possible way to understand the relationship between inflammation and neuropsychological functioning versus the standard approach of examining biomarkers individually. 

## Figures and Tables

**Table 1:** Coefficient of variation by marker.

Marker	Combined	AD	Control
TNFalpha	58.18	54.55	59.68
A1A	49.24	44.44	53.13
IL8	11.38	10.29	12.21
CRP	190.02	317.43	139.39
IL-1ra	15.04	15.54	14.15
IL10	31.55	27.96	32.98

A1A: alpha-1 antitrypsin; CRP: C-reactive protein.

**Table 2:** Demographics by diagnosis.

	AD	Control	*P* value
Number of subjects	124	196	—
Sex			
Female	77 (62.1%)	134 (68.4%)	0.25
Age (years)			
Mean (SD)	76.72 (7.80)	70.47 (8.81)	<0.01
Range	57–91	55–90	
Education (years)			
Mean (SD)	14.35 (3.30)	15.52 (2.73)	<0.01
Range	5–22	10–25	
ApoE			
One or more E4	71 (61.7%)	50 (25.8%)	<0.01

**Table 3:** Neuropsychological testing by diagnosis.

	AD	Control	*P* value
Number of subjects	124	196	—
MMSE			
Mean (SD)	22.32 (4.13)	29.43 (0.88)	<0.01
Range	8–30	26–30	
CDR sum of boxes			
Mean (SD)	5.15 (2.30)	0.00 (0.04)	<0.01
Range	0.5–13.0	0.0–0.5	
Trails A			
Mean (SD)	6.89 (2.86)	10.31 (2.68)	<0.01
Range	2–14	2–17	
Trails B			
Mean (SD)	5.27 (3.42)	10.96 (2.53)	<0.01
Range	2–18	3–18	
WMS-III Logical Memory I			
Mean (SD)	4.64 (2.46)	13.57 (2.76)	<0.01
Range	1–10	6–18	
WMS-III Logical Memory II			
Mean (SD)	3.69 (1.89)	13.99 (2.63)	<0.01
Range	1–11	5–19	
WMS-III Visual Reproduction I			
Mean (SD)	4.97 (2.77)	12.41 (3.19)	<0.01
Range	1–13	5–18	
WMS-III Visual Reproduction II			
Mean (SD)	4.82 (2.27)	13.57 (3.14)	<0.01
Range	2–12	5–19	
Boston naming test			
Mean (SD)	7.32 (3.79)	11.94 (3.04)	<0.01
Range	2–16	2–17	
COWAT			
Mean (SD)	7.91 (2.89)	11.64 (2.75)	<0.01
Range	2–18	4–18	
AMNART			
Mean (SD)	9.28 (3.55)	12.11 (3.37)	<0.01
Range	2–18	2–18	
Estimated VIQ			
Mean (SD)	108.32 (15.05)	117.12 (9.88)	<0.01
Range	38.66–127.44	42.11–132.28	

**Table 4:** Biomarkers by diagnosis.

	AD	Control	*P* value
Number of subjects	124	196	—
TNF-alpha Log			
Mean (SD)	0.55 (0.30)	0.62 (0.37)	0.05
Range	−0.30–1.04	−0.30–1.79	
Alpha-1 Antitrypsin Log			
Mean (SD)	0.36 (0.16)	0.32 (0.17)	0.09
Range	−0.23–0.71	−0.43–0.71	
IL-8 Log			
Mean (SD)	1.36 (0.14)	1.31 (0.16)	<0.01
Range	1.04–1.78	0.98–1.84	
C-Reactive Protein Log			
Mean (SD)	0.14 (0.52)	0.33 (0.46)	<0.01
Range	−1.34–1.36	−1.19–1.40	
IL-1ra Log			
Mean (SD)	1.93 (0.30)	2.05 (0.29)	<0.01
Range	0.90–2.61	0.90–2.62	
IL-10 Log			
Mean (SD)	0.93 (0.26)	0.94 (0.31)	0.83
Range	0.04–1.43	0.04–1.62	
Sum of markers			
Mean (SD)	2.40 (1.85)	2.76 (1.95)	0.11
Range	0–8	0–8	
